# Pediatric Drug-Resistant Tuberculosis: The Current and Future Prospects for Management and Prevention

**DOI:** 10.3390/pathogens12111372

**Published:** 2023-11-20

**Authors:** Dhanya Dharmapalan, Sushant Satish Mane

**Affiliations:** 1 Apollo Hospitals, Navi Mumbai 400614, India; drdhanyaroshan@gmail.com; 2 Sir JJ Group of Hospitals, Grant Govt. Medical College, Mumbai 400008, India

**Keywords:** bedaquiline (BDQ), delamanid (DLM), Xpert MTB, tuberculosis, TB infection, latent TB, TB vaccines, TB preventive treatment

## Abstract

In the continued battle against one of the oldest enemies known to mankind, *Mycobacterium tuberculosis (MTB)*, the emergence of drug resistance to antituberculosis drugs among children poses multiple challenges for early detection and treatment. Molecular diagnostics and newer drugs like bedaquiline and delamanid have strengthened the armamentarium and helped design convenient, safe, and child-friendly therapeutic regimens against drug-resistant tuberculosis (TB). Preventive strategies like treatment of TB infection among children living in close contact with patients with drug-resistant TB and effective vaccines against TB are currently in the investigative stages of development and implementation. In addition to the implementation of recent novel diagnostics and treatment modalities, effective psychosocial and nutritional support, as well as dedicated monitoring for compliance and adverse effects, are crucial determinants for successful treatment outcomes in these children.

## 1. Introduction

Drug-resistant tuberculosis (DRTB) has posed a great challenge to the detection and management of tuberculosis in children, especially those less than 15 years of age. A child is diagnosed with DR TB after navigation of a sequence of complex steps, including accurate clinical suspicion, appropriate clinical and radiological testing, effective specimen collection, and whenever possible, microbiological confirmation. Each of these steps has its own set of challenges among children. The clinical clues of chronic cough need to be differentiated from prolonged cough in post-viral illnesses and the hyperactive airway diseases that are common among children. A long list of differentials for inadequate weight gain or recurrent abdominal pain requires the clinician to be able to narrow down the differential diagnosis to suspect TB by proper clinical examination. Families may hide a history of TB contact to avoid the stigma associated with the disease. Tests such as tuberculin sensitivity test (TST) and interferon-gamma release assays (IGRAs), if available, cannot distinguish definitively between TB infection and TB disease, and negative tests do not necessarily rule out TB. Chest X-rays (CXRs), when available, remain the primary imaging tool for pediatric pulmonary TB, particularly in high TB-burden countries, where access to more advanced imaging is limited. Although the radiological disease findings on CXR in children are wide, CXRs can serve as a critical component, when available, of the child TB diagnostic approach and can be used to support clinical decision-making [1].

Due to the paucibacillary nature of pediatric TB, the next step of obtaining a good-quality specimen is another difficult task. However, microbiological confirmation should always be sought whenever possible, using available and recommended diagnostic tests and appropriate pediatric specimens, such as sputum, gastric aspirates, nasopharyngeal aspirates (NPA), stool, and urine samples. Despite the best efforts and most advanced techniques, the microbiological positivity rate in detecting *Mycobacterium tuberculosis* (MTB) might not approach more than 30–40% [2]. It is only within this subset of pediatric patients with positive microbiological tests that the concept of ‘universal DST’ can be applied (i.e., confirmation of resistance to at least rifampicin).

For children with DR TB, there is therefore another large subset of the population that will be diagnosed clinically and referred to as having “presumptive” or “probable” DR TB. These children are those that will likely then be treated empirically for DR TB based upon clinical history and presentation, the results of non-microbiological tests including imaging results, and a history of exposure to close contact with a DR TB index case or poor response/failure to treatment with the first-line antituberculosis drugs (i.e., isoniazid, rifampicin, pyrazinamide, and ethambutol).

Due to the clinical and diagnostic challenges outlined, the annual figure of 30,000 new pediatric DR TB cases reported globally seems likely to be just the tip of the iceberg, while a large number of children remain undiagnosed and, therefore, do not receive the appropriate curative treatment [3].

Despite the growing burden of multidrug resistance and the challenges in diagnosing and treating children with DR TB, there have been significant advances in both the diagnostic and therapeutic modalities available to children with TB, offering some light at the end of the tunnel in our fight against pediatric DR TB.

## 2. Molecular Tests—The Game Changers in TB Diagnostics

It is challenging to confirm drug resistance in TB through clinical precision and radiological methods. Therefore, clinicians should depend whenever possible on microbiological techniques; however, starting empiric DR TB treatment in a child should not be delayed while awaiting microbiological confirmation. Delay in diagnosis of drug resistance can lead to improper treatment, clinical worsening for the child, and, also, increased opportunity of transmission of TB. Liquid cultures have reduced the turnaround time for isolating MTB compared with the solid media cultures to as early as two weeks and are the ‘gold standard’ for the diagnosis. Yet, cultures are fraught with the disadvantage of the slow growth of MTB. Time is important in identifying the complete drug resistance profiles of the causative MTB pathogen as soon as possible to guide and tailor treatment decisions.

Chromosomal mutations in the existing genes in MTB are passed vertically from mother to daughter cells to confer drug resistance [4]. Examples of these resistance genes are isoniazid (alterations in genes *katG* and *inhA)*; rifampicin (in *rpoB*); streptomycin (in *rrs* and *rpsL*); pyrazinamide (in *pncA*); ethambutol (in *embB*); quinolones (in *gyrA*); and kanamycin (in *rrs*) [5]. Unlike other bacteria, these cells do not have plasmids and rarely exchange DNA laterally.

The following molecular diagnostics, which help in target amplification and gene sequencing of these known or suspected resistance genes, have been recently heralded as game changers for rapid diagnosis and initiation of appropriate treatment of DR TB:

### 2.1. Xpert MTB/RIF Assay

Xpert MTB/RIF assay is a fully automated nucleic acid amplification test and a cartridge-based assay that was approved for use by the WHO in 2010. It provides the diagnosis of MTB and rifampicin resistance in two hours. It involves a cartridge containing a sputum sample mixed with a reagent available with the assay. It detects DNA from both live and dead bacteria. The results for rifampicin resistance are interpreted as detected or “not detected or indeterminate.” An indeterminate result means it is not possible to verify by the assay the status of resistance of MTB to antituberculosis drugs.

### 2.2. Xpert Ultra (Cepheid Inc., Sunnyvale, CA, USA) 

Xpert Ultra is an automated closed system that works on the same GeneXpert platform as Xpert MTB/RIF. The results are available in a shorter time of 80 min, about 40 min earlier than the Xpert MTB/RIF assay. The sensitivity of Xpert Ultra is higher than that of Xpert MTB/RIF due to its lower limit of detection (15 colony-forming units (CFU)/mL which is 1-log lower than that of Xpert MTB/RIF (112 CFU/mL)) [6,7]. Like the Xpert MTB/RIF assay, it detects DNA of both live and dead MTB. The melting temperature analysis in Xpert Ultra, instead of real-time PCR analysis with Xpert MTB/RIF, has also improved the detection of rifampicin resistance, especially in mixed infections. This improved performance of the Ultra assay is due to the ability to differentiate between resistance-conferring mutations and silent mutations such as Q513Q and F514F [8]. However, its ability to detect very low MTB loads gives rise to trace positive reports and the inability to determine rifampicin resistance.

### 2.3. Xpert MTB/XDR Assay

This assay helps in identifying mutations associated with resistance to isoniazid, rifampicin, ethionamide, fluoroquinolones, and second-line injectables like amikacin, kanamycin, and caperomycin. Although the assay does not detect resistance to newer drugs such as bedaquiline (BDQ) and delamanid (DLM), it helps in the detection of 97% of current MDR TB in the adult population. Data on children are limited. However, its full utility may be diminished due to the recent move from injectable drugs to all oral TB drug regimens for eligible adults and children [9].

### 2.4. Line Probe Assays

MTBDRplus line probe assay is based on strip technology involving three steps of DNA extraction, amplification of multiplex PCR, and reverse hybridization. It is used for the rapid diagnosis of MTB and resistance to RIF and INH. It is performed as per the instructions of the manufacturer (Hain LifeScience GmbH, Nehren, Germany). MTBDRplus can only detect mutations in *rrs,eis* genes, *gyrA*, and *gyrB* genes; therefore, additional phenotypic testing is required for a complete resistance profile.

### 2.5. Whole Genome Sequencing (WGS)

WGS provides DNA sequence data for the entire tuberculosis genome. It is a very promising tool as it overcomes the disadvantage of the limited number of known mutations that can be detected by molecular diagnostic tests. WGS helps in identifying resistance to newer TB drugs like BDQ, DLM, and linezolid and provides additional data to understand demonstrated phenotypic resistance. Also, the turnaround time for WGS is a few days compared with the several weeks required for drug susceptibility tests on MTB cultures. WGS is reported to have higher sensitivity and specificity in screening resistance in the first-line TB drugs than in the second-line drugs [10]. It can also help in differentiating new TB infections and disease and TB disease relapse, mapping the transmission network and tracing the source case. There are several challenges for WGS related to low DNA yield when sequencing directly from sputum, required expertise for analysis of large sequence data standardizations, and cost. WGS data in the pediatric age group are sparse.

### 2.6. CRISPR-MTB

The clustered regularly interspaced short palindromic repeats assay (CRISPR) can work like molecular scissors on MTB and is a promising tool that is being evaluated in children as it can detect MTB directly from all bodily fluids with great sensitivity. It uses the technology of polymerase-mediated DNA amplification as well as *Cas12a*-mediated enzymatic signal amplification. The sample size required is very small, about 500 μL irrespective of the type of sample. This assay takes less time than Gene Xpert and is completed in 1.5 h. A diagnostic sensitivity and specificity of over 90% was reported for MTB detection from blood specimens of a pooled group of adults and children [11]. An expanded CRISP-MTB assay can also help in detecting resistance to TB drugs [12].

### 2.7. Application of Molecular Tests with Improved Diagnostic Approaches in Children

#### 2.7.1. Induced Sputum

Induced sputum is a noninvasive method of sputum collection with a microbiological yield similar to three sequential gastric lavages [13]. The steps involved are inhalation of salbutamol followed by nebulization with hypertonic saline. This is followed sequentially by percussion of the chest resulting in spontaneous expectoration of sputum by the child. This method can be performed in an ambulatory setting with a minimum of four hours of sample and is, therefore, more convenient than the invasive gastric aspirate sample collection that requires overnight fasting. The sensitivity of Xpert MTB/RIF on induced sputum in children is about 66% with a specificity of 98% in comparison to culture [14].

#### 2.7.2. Nasopharyngeal Aspirate

Nasopharyngeal aspirates are another type of relatively noninvasive specimen collection in children. After 2 h of fasting, the nasopharynx of the child, who is in the supine position, is suctioned using a sterile catheter inserted in the nostril. This catheter has either a mucus trap or a suctioning device. Some children may find the procedure unpleasant [15]. Like induced sputum, this method can be performed in an ambulatory setting. The pooled sensitivity and specificity of Xpert MTB/RIF on NPA reported by a systematic review and meta-analysis were 65.2% and 97.9%, respectively compared with a reference standard using either the specimens of sputum or gastric lavage [15].

#### 2.7.3. Stool

MTB bacilli are known to be shed in the stool after overnight swallowing of sputum containing MTB. The feasibility of stool testing methods provides for the convenience of noninvasive specimen collection, especially in children younger than five years, who currently often require more invasive TB diagnostic methods. The pooled sensitivity of Xpert MTB/RIF testing on stool specimens was higher in children with HIV compared with children uninfected with HIV in two independent systematic reviews and meta-analyses that analyzed the diagnostic accuracy of Xpert testing of stool specimens for diagnosis of pulmonary TB [16,17,18]. The authors noted the limitations of the lack of age-specific data on performance, especially for the younger age groups, as well as the wide variability of stool preparation and testing protocols. A study conducted in India on stool samples of patients with extrapulmonary TB, using clinically/microbiologically confirmed TB as the reference, showed a very high specificity of about 95% and the highest sensitivity of about 73.5% from lymph node TB, followed by abdominal TB, and lowest of 56.3% for pleural effusion. In the same study, the sensitivity of stool PCR was about 97.2% for smear-positive pulmonary TB patients and almost 77% for those with smear-negative TB [19].

In comparison with Xpert MTB/RIF, Xpert Ultra testing of stool specimens had higher sensitivity and lower specificity [20]. The higher sensitivity was attributed to the low limit of detection and ability to detect trace positive results. The current methods used for processing stool specimens for such tests are relatively resource-intensive. Due to the high percentage of trace positive results in Xpert Ultra, further comparative evaluation is required for diagnostic accuracy in children with and without trace positive results.

The WHO guidance has recommended stool specimens alongside specimens like induced sputum, nasopharyngeal aspirate, or gastric aspirate using either Xpert MTB/RIF and Xpert Ultra for diagnosis of pulmonary TB in children under 10 years of age since 2021 [21]. A practical manual for processing stool samples was released in 2022 to standardize the testing methodology of stool specimens for TB testing tuberculosis [22].

#### 2.7.4. Tissue Biopsy and CSF

Children have a higher proportion of extrapulmonary TB compared with adults. Xpert MTB/RIF utilized to test lymph node (LN) tissue or LN aspirates and the cerebrospinal fluid has a relatively lower sensitivity of 80% and 42%, respectively, despite high specificity of over 90% [17]. In a pediatric study of 55 cases of TB meningitis, the Xpert MTB/RIF assay demonstrated a sensitivity and specificity of 40% and 92.5%, respectively, and a 78.2% diagnostic accuracy compared with culture [23].

#### 2.7.5. Combination of Two or More Specimens for Molecular Testing Methods

A Cochrane review of Xpert MTB/RIF Ultra in children suggests that the use of molecular methods in sputum, gastric aspirate, and NPA is an accurate method for detecting pulmonary TB and rifampicin resistance in children; they found the highest sensitivity for sputum, followed by gastric aspirate and stool, and the lowest sensitivity with nasopharyngeal aspirate [24]. Duplication of nasopharyngeal aspirate or one nasopharyngeal aspirate plus one stool specimen is reported to produce a good microbiological yield tested by Xpert MTB/RIF assay or MGIT culture method with sensitivity above 70% in young children and equivalent to the yield of two samples of gastric aspirate or two samples of induced sputum [25]. In this study, the sensitivity was calculated as the number of children with positive test results from the candidate index combination divided by the number of children with positive test results on Xpert MTB/RIF assay/culture for at least one primary study specimen [25]. Novel and less invasive upper respiratory samples like oral and laryngeal swabs hold promise for further investigations of alternative methods for sampling in children [15].

## 3. New Players in TB Therapeutics in Children

The pharmacokinetic data of the antituberculosis drugs in children are limited, and most of the doses used in the current regimens are extrapolated from weight-based adult doses. The restricted availability of pediatric-friendly drug formulations also creates issues with compliance [26].

Delayed diagnosis, limited psychosocial and nutritional support, incomplete monitoring of adverse events, and incomplete coordination with other programs, such as HIV and nutrition, as well as incompletely trained staff, are common problems that are faced in resource-limited settings.

Therefore, it is no surprise that there is a striking difference in the pooled treatment success of MDR TB with the present recommended regimens among children belonging to LMI countries (73%) in comparison to those in developed nations (87%) [27].

### 3.1. Clinical Pharmacological Studies in Children

Due to the age-related differences and changes in pharmacokinetic and pharmacodynamic (PK/PD) parameters in children, the pediatric doses derived from extrapolation from adults are inaccurate and therefore inappropriate [28]. PK/PD studies will help to identify the dosing regimen that can safely expose children to the therapeutic drug levels that adults achieve with their standard recommended doses [28]. Also, there is a paucity of prospective studies on the adverse effects of antituberculosis drugs in children. Adverse effects such as hearing and vision impairment or neuropathy might be underreported due to the difficulty in diagnosing these impairments without specialized tests. MDRPK1 and MDRPK2 studies (observational clinical trials for children in Cape Town, South Africa) were conducted in 2011–2015 and 2016–2018, respectively, to close the knowledge gaps for PK and safety data for second-line antitubercular agents. In MDR-PK1, PK data were obtained for ethionamide, terizidone, and para-aminosalicylic acid (PAS), while MDR-PK2 data helped in optimizing the dosing regimens of levofloxacin, moxifloxacin, and linezolid in young children [29,30,31]. The studies found that moxifloxacin doses need to be increased above the currently recommended 10–15 mg/kg/day in young children to be equivalent to the therapeutic adult doses. However, safety issues have not been evaluated, especially when co-administered with other drugs that prolong the QT interval [31].

Novel drug formulations of second-line drugs, such as dispersible tablets for bedaquiline, delaminid, pretomanid, and gummy formulations for moxifloxacin and clofazamine are being developed for better palatability and acceptance [28].

### 3.2. New and Repurposed Drugs

In March 2022, the WHO recommended the use of BDQ and DLM in children of all age groups, for treatment of drug-resistant TB [21], and in 2023, they also released two information notes to provide practical information and guidance for the implementation of novel formulations of BDQ and DLM in line with recommendations for their use in children of all ages [32,33]. These new and repurposed drugs have brought promising opportunities to shorten the otherwise long duration of treatment and to move away from injectable drugs and drugs with severe adverse effects and drug interactions.

#### 3.2.1. Delamanid (DLM)

DLM inhibits the synthesis of methoxy- and keto-mycolic acid present in the cell wall of MTB while generating nitrous oxide. It has an oral bioavailability of up to 47%, and peaks at around 4–8 h after oral dosing with a half-life of 30–38 h. A steady-state concentration is reached after about 2 weeks [34]. DLM is generally well tolerated. However, due to its potential adverse effect of prolongation of QT interval, clinicians are advised to monitor for cardiac safety with ECG and serum electrolytes and, also, to avoid other drugs that can prolong the QT interval. Several ongoing clinical trials evaluating the safety of DLM in children (including NCT01859923, NCT01856634, and NCT03141060) are in the early phases [35].

Based on the growing evidence of efficacy at the time of making the recommendation, the WHO lowered the age recommendation for DLM from above 6 years in 2016 to include 3 to 6 years of age in 2019 and, subsequently, to all ages for inclusion into longer duration treatment regimens [21]. It should be noted that the adult formulation (50 mg) of demand is not to be split or crushed for dosing in pediatrics as the bioavailability can be compromised; therefore, the 25 mg dispersible formulation needs to be used in children.

#### 3.2.2. Bedaquiline (BDQ)

BDQ inhibits the mycobacterial ATP synthase proton pump. It reaches peak serum concentration in 5–6 h and has a half-life of more than 24 h. BDQ is usually well tolerated with common side effects of nausea, headache, and arthralgia. It can cause prolongation of QT interval (especially after 18 weeks of use) and, therefore, should be used with caution with DLM, clofazimine, and fluoroquinolones, which have similar adverse effects. It should be noted that its efficacy can be reduced when it is co-administered with *CYP3A* inducers like rifampicin, while its toxicity can be increased when it is administered with ketoconazole or lopinavir/ritonavir [36].

Interim reports were available from the WHO, from ongoing pediatric trials on BDQ, namely TMC207-C211 in children aged 5–18 years and IMPAACT P1108 in children aged 0–6 years in 2022, which did not note any higher adverse effects in the pediatric age group in comparison to the adults [21,37], (see Table 1, Table 2 and Table 3). After extrapolating the data on efficacy from the adult population, the WHO stated that:

“*A shorter all-oral bedaquiline-containing regimen of 9–12 months duration is recommended in eligible patients with confirmed multidrug- or rifampicin-resistant tuberculosis (MDR/RR-TB) who have not been exposed to treatment with second-line TB medicines used in this regimen for more than 1 month, and in whom resistance to fluoroquinolones has been excluded*.”

A mutant *atpE* gene can confer resistance to BDQ. Currently, there is no standard way of testing for BDQ resistance [38].

Current recommended regimens by the WHO for DR TB treatment in children are summarized in Table 1.

In children with rifampicin-sensitive and isoniazid-resistant TB, treating with rifampicin, ethambutol, pyrazinamide, and levofloxacin for 6 months is recommended. In the children eligible for the 9-month all-oral drug regimen, BDQ is given for 6 months with a combination of levofloxacin/moxifloxacin, ethionamide, ethambutol, high-dose isoniazid, pyrazinamide, and clofazimine for at least 4 months, with extension to 6 months in case of sputum positivity at 4 months. This is followed by a 5-month regimen of levofloxacin/moxifloxacin, clofazimine, ethambutol, and pyrazinamide for 5 months [21].

For children who do not meet the eligibility criteria for the standardized all-oral BDQ regimen, it is recommended to provide longer and individualized treatment regimens containing BDQ and at least other four medicines to which the organism is susceptible. Priority needs to be given to Group A and Group B medicines in determining the regimen with the addition of Group C drugs based on the assessment of risks and benefits [21].

## 4. Prevention of TB and DR TB

As per mathematical modeling, three in every 1000 people carry latent DR TB, and children below 15 years of age have double the prevalence of latent TB [39]. There are limited observational studies involving preventive therapy in children who are in contact with MDR household members.

### 4.1. TB Preventive Therapy (TPT)

TB preventive therapy will prevent the progression from DRTB infection to DRTB disease. The most vulnerable to such a progression are the household members of DRTB patients [40]. There are currently several ongoing trials for MDR TB preventive therapy in children. TheV-QUIN Phase III in Vietnam and TB CHAMP Phase III in South Africa are evaluating the efficacy of levofloxacin versus placebo as TPT. The PHOENix Phase III compares the efficacy of delaminid versus isoniazid as part of an international multi-center trial. All three trials are evaluating TPT treatments of 6-month duration with follow-up for up to 2 years [41]. It was found in the lead-in study to the TB CHAMP trial that although the novel dispersible tablet of 100 mg levofloxacin was acceptable, there remained the challenges of a psychosocial burden on caregivers for providing effective preventive therapy to children [42]. Current WHO guidelines recommend that preventive therapy be considered for children in contact with patients with MDR-TB. The decision needs to be based on individualized risk assessment and sound clinical justification [43]. The WHO recommends using a fluoroquinolone-based regimen with or without a companion drug as per tolerance [43]. Studies are lacking on fluoroquinolone-resistant MDR TB contacts [43].

### 4.2. TB Vaccines

In the year 2021, the Bacillus Calmette–Guérin vaccine (BCG) “celebrated” its 100th anniversary. The WHO’s Expanded Program on Immunization (EPI) has included BCG since 1976 and to date more than 4 billion people have received the vaccine, the majority children. BCG is given to newborns and is included in the national immunization schedules in 154 countries, with coverage rates of >85% [44]. However, protection from BCG has its limits. The protective efficacy of BCG is reported as 60–80% against severe forms of tuberculosis (including TB meningitis and miliary TB) in children. It is estimated to protect against TB infection in about 20 percent of children and likely to protect 50% infected from developing active TB disease. The protection for adolescents and adults has been poor [45]. BCG was found to be highly effective in the absence of prior MTB or sensitization of mycobacteria from the environment [46].

The WHO has developed preferred product characteristics (PPC) for safer and more effective TB vaccinations in infants. The vaccine that has been prioritized as most needed is the one that can be provided to adolescents and adults for the prevention of TB. Such a vaccine would help break the community transmission of TB.

The pipeline for vaccines against TB consists of whole-cell vaccines, recombinant subunit vector vaccines, and adjuvant proteins among the 14 candidates. These vaccines are currently in clinical development (phase 1 and beyond) [47,48] and are being evaluated for their use in the prevention of disease (POD), prevention of infection (POI), and prevention of recurrence (POR).

At present three candidates are in phase 3 development: VPM1002, MIP, and MTBVAC. VPM1002 and MIP. The safety and efficacy trials are being conducted among healthy household contacts >6 years of age of newly diagnosed sputum-positive pulmonary TB patients [49]. VPM1002 and MTBVAC are being evaluated for POI among newborns and POD among infants, respectively [50,51].

A POI vaccine, which can be given at birth, could be the ideal candidate as protection would be conferred earlier in life. However, given the potential impact a POD vaccine can have if this vaccine is given to older children, adolescents, and adults under programmatic settings, it is being funded as an initial TB vaccine rollout candidate. Vaccines providing both POD and POI have the benefits of protecting not only infants from birth but also older children and adolescents from developing TB disease if they have already been exposed to TB.

## 5. Challenges and the Way Forward

It is encouraging to note that children with XDR TB have had much more favorable outcomes in comparison with adults [52]. Every effort needs to be made to ensure that each child gets the appropriate management customized to the pathogen resistance pattern while minimizing adverse events. Decentralization of TB services is recommended through peripheral family-centered integrated care services [21]. Capacity building for prompt diagnosis and treatment, continuous training of health care staff, provision of regular supplies of TB medicines, monitoring for adverse effects and compliance, and additional support systems for nutrition and psychological support, coupled with attention to contact tracing and abolishing the stigma associated with TB are crucial for the successful outcome of DR TB treatment.

## Figures and Tables

**Table 1:** Current regimens recommended by WHO for DR TB treatment in children [21].

Regimens	Regimen	Eligibility
Isoniazid monoresistance	6-month regimen of (H)REZ-Lfx. In case Lfx cannot be used, (H)REZ to be given for 6 months. No need to add streptomycin.	For children of any age with isoniazid monoresistance.
Shorter all-oral 9-month regimen for MDR/RR-TB	4–6 months regimen of Bdq _(6 months)_-Lfx/Mfx-Cfz-Z-E-Hh-Eto /5-month regimen of Lfx/Mfx-Cfz-Z-E	MDR/RR TB fluoroquinolone resistance excluded Not exposed to second-line TB medicines for more than one month No extensive TB disease+ No severe extrapulmonary TB
Shorter regimen for MDR/RR-TB with quinolone resistance	6–9 month treatment regimen composed of bedaquiline, pretomanid, and linezolid—BPaL regimen *	Bacteriologically confirmed pulmonary TB At least 14 years of age and weight greater than 35 kg Not pregnant/breastfeeding and willing to use effective contraception No known allergy/known resistance to any components of the regimen Not exposed to components for more than 2 weeks No extrapulmonary TB Relative contraindications: Concurrent use of medications that can have drug interaction with BPaL component drugs High risk of cardiac arrhythmia Severe anemia, leucopenia, or thrombocytopenia Severe hepatic failure Severe renal failure Severe neuropathy
Longer regimen for MDR/RR-TB	18-month regimen Bdq_(6 m)_-Lfx/Mfx-Lzd-Cfz	For those not eligible for a shorter all-oral bedaquiline-containing MDR TB regimen

* Moxifloxacin can be added to this regimen (BPaLM) in case there is an absence or unknown resistance to fluoroquinolones. H: isoniazid, Hh: high-dose isoniazid, R: rifampicin, E: ethambutol, Z: pyrazinamide, Lfx: levofloxacin, Bdq: bedaquiline, Mfx: moxifloxacin, Cfz: clofazamine, Eto: ethionamide, Pa: pretomanid. +Extensive TB disease is defined in this document as the presence of bilateral cavitary disease or extensive parenchymal damage on chest radiography. In children under 15 years of age, the advanced disease is usually defined by the presence of cavities or bilateral disease on chest radiography. + Severe extrapulmonary TB is defined as the presence of miliary TB or TB meningitis. In children under 15 years of age, extrapulmonary forms of disease other than lymphadenopathy (peripheral nodes or isolated mediastinal mass without compression) are considered severe.

**Table 2:** Regimen for bedaquiline, pretomanid, and linezolid (BPaL) for ages ≥ 14 years * [21].

Antitubercular Drug	Dose
Bedaquiline	400 mg OD × 2 weeks, then 200 mg 3 times per week
Pretomanid	200 mg OD
Linezolid	1200 mg OD (dose can be reduced in case of linezolid-induced neuropathy)

* Treatment duration is 6–9 months.

**Table 3:** Drug doses and side effects for second-line drugs based on estimated therapeutic efficacy (adapted from WHO recommendations) [21].

Drug	Dosage	Major Side-Effect
Levofloxacin	15–20 mg/kg	QT prolongation, psychiatric disturbance
Moxifloxacin	10–15 mg/kg 10 mg/kg in less than 6 months	QT prolongation, psychiatric disturbance
Bedaquiline	For 100 mg tablet: (100 mg in 10 mL = 10 mg/mL) 0 to <3 months: 3 mL OD for 2 weeks; then 1 mL OD M/W/F for 22 weeks ≥3 to <6 months (3 to <10 kg): 6 mL OD for 2 weeks; then 2 mL OD M/W/F for 22 weeks ≥6 months (10 to <16 kg): 8 mL OD for 2 weeks; then 4 mL OD M/W/F for 22 weeks 16–30 kg: 2 tab OD ×2 weeks then 1 tab OD M/W/F for 22 weeks 30 to <46 kg: 4 tab OD × 2 weeks then 2 tablet OF M/W/F for 22 weeks	Drug interactions with drugs that inhibit or induce cytochrome P450 enzymes, QT prolongation
	For 20 mg dispersible tablet: 0 to <3 months: 1.5 OD for 2 weeks; then 0.5 OD M/W/F for 22 weeks ≥3 to <6 months: 3 OD for 2 weeks; then 1 OD M/W/F for 22 weeks ≥6 months (7 to 10 kg): 4 OD for 2 weeks; then 2 OD M/W/F for 22 weeks ≥6 months (10 to <16 kg): 6 OD for 2 weeks; then 3 OD M/W/F for 22 weeks 16–29 kg: 10 OD for 2 weeks then 5 tablet OD M/W/F × 22 weeks. >29 kg: 20 OD × 2 weeks then 10 DTS M/W/F × 22 weeks.	
Linezolid	For 1–15 kg 15 mg/kg OD; For >15 mg/kg 10–12 mg/kg OD	Bone marrow suppression, peripheral neuropathy, optic neuritis, gastrointestinal disorders
Clofazamine	2–5 mg/kg (give on alternate days if the daily dose is very high)	Can prolong QT when used with drugs that prolong QT like BDQ, DLM, and fluoroquinolones Orange discoloration of skin, conjunctiva, cornea, and body fluids; dry skin, pruritus, rash, ichthyosis, and xerosis; gastrointestinal intolerance; and photosensitivity Dose adjustment needed in severe hepatic insufficiency
Cycloserine	15–20 mg/kg	Inability to focus, weakness, depression, psychosis and suicidal ideation, seizures, peripheral neuropathy, lichen planus, and Stevens–Johnson syndrome.
Ethambutol	15–25 mg/kg	Ophthalmic, GI disturbance
Delamanid	<3 months: 25 mg OD ≥3 months: <16 kg: 25 mg BD; 16 kg to <30 kg: 50 mg morning and 25 mg evening; >30 kg: 50 mg BD 12–17 years:100 mg BD	Nausea and vomiting, QT prolongation, hallucinations, paraesthesia
Pyrazinamide	30–40 mg/kg	Hepatotoxicity, arthralgia, GI disturbance, dermatological disorder
Meropenem	20–40 mg/kg/IV every 8 hourly (to be used with Clavulanic acid)	GI disturbances, seizures, hepatic and renal dysfunction
Amikacin	15–20 mg/kg/day (Max 1 g/day)	Nephrotoxicity, ototoxicity
Streptomycin	20–40 mg/kg (Max 1 g/day)	Ototoxicity
Ethionamide or prothionamide	15–20 mg/kg (Max 1 g/day)	Hypothyroidism
P-amino salicylic acid	200–300 mg/kg in 2 divided doses	Hypothyroidism, GI disturbance
Isoniazid	15–20 mg/kg/dose (To be given with pyridoxine)	Peripheral neuropathy

## Data Availability

Not applicable.
